# Nationwide analysis of mortality predictors following in-hospital cardiac arrest in older Thai adults, 2016–2024

**DOI:** 10.1016/j.resplu.2026.101282

**Published:** 2026-02-25

**Authors:** Manchumad Manjavong, Panita Limpawattana, Jarin Chindaprasirt, Watsamon Inyu, Poonchana Wareechai

**Affiliations:** a Division of Geriatric Medicine, Department of Internal Medicine, Faculty of Medicine, Khon Kaen University, Khon Kaen 40002, Thailand; b Division of Oncology, Department of Internal Medicine, Faculty of Medicine, Khon Kaen University, Khon Kaen 40002, Thailand; c Division of Critical Care, Department of Internal Medicine, Faculty of Medicine, Khon Kaen University, Khon Kaen 40002, Thailand; d National Health Security Office, Bangkok 10210, Thailand

**Keywords:** Aging, Cardiopulmonary resuscitation, Geriatric, Heart arrest, Survival rate

## Abstract

**Background:**

In-hospital cardiac arrest (IHCA) is common among older adults, but recent detailed studies in Thailand on trends or factors related to hospital mortality in this group are lacking. This study aimed to analyze trends in in-hospital cardiopulmonary resuscitation (CPR) among older adults, their outcomes, and survival factors to hospital discharge using the national database from 2016 to 2024.

**Methods:**

National data from Thailand’s National Health Security Office covered all inpatients aged ≥60 years who received IHCA during 2016–2024, defined by the International Statistical Classification of Diseases and Related Health Problems, 10th Revision, Thai Modification (ICD-10-TM) based on ICD-9-99.6. Retrospective data from discharge summaries examined age-adjusted prevalence and survival rates of older CPR patients, normalized to 100,000 Thais, focusing on survival after CPR and associated factors of discharge mortality.

**Results:**

A total of 190,271 older patients received CPR. The most common primary diagnosis was cardiovascular disease (41.4%). Age-adjusted CPR rates were 221/100,000 males and 147/100,000 females. The survival rate to hospital discharge was 33%, with age-adjusted rates of 72/100,000 males and 50/100,000 females. Mortality was linked to male gender (adjusted odds ratio [AOR] 1.06), older age (AOR 70–79: 1.11; over 80: 1.30), specific diagnoses, arrest types (AOR for non-shockable 1.21; shockable 0.39–0.69), and procedures like coronary artery bypass Grafting (AOR 0.21), intra-aortic balloon pump (AOR 2.05), pacemaker (AOR 0.24), extracorporeal membrane oxygenation (AOR 5.18), and mechanical ventilation ≥96 h (AOR 0.88).

**Conclusions:**

CPR rates were higher in older males than in females. Cardiovascular disease was the main primary diagnosis. Overall survival to hospital discharge was about 33%. Mortality was linked to male sex, older age, cardiac rhythm types, diagnoses, and procedures.

## Highlights

• CPR rates peaked in males & older adults but dropped in 2020.
• Survival improved to 33%, better than previous Thai data.
• Mortality associated with sex, age, rhythm type, diagnoses, and interventions.

## Introduction

In-hospital cardiac arrest (IHCA) is common among older adults, who constitute a large proportion of IHCA patients.^1^, ^2^, ^3^, ^4^, ^5^ The occurrence rate is estimated at 3–8 per 1000 hospital admissions, increasing with age and the presence of comorbidities.^1^, ^2^, ^3^, ^4^, ^5^ A large population-based sample in South Korea, comprising 298,676 patients, reported that 60.7 per 100,000 adults experienced in-hospital cardiopulmonary resuscitation (CPR) in 2016, up to 92.1 per 100,000 adults in 2019.^2^ This might be due to population aging and variations in hospital admission practices. Among this population, the primary causes of IHCA are cardiovascular diseases, respiratory failure, and sepsis. Usually, non-shockable rhythms, such as asystole and pulseless electrical activity (PEA), are most common, indicating underlying chronic conditions or hypoxia-related issues.^6^, ^7^, ^8^, ^9^ Although shockable rhythms such as ventricular fibrillation (VF) and pulseless ventricular tachycardia (VT) are less frequent, they generally have better outcomes.^10^

Overall survival to hospital discharge among elderly IHCA patients worldwide generally ranges from 18.7% to 28.5%.^2^, ^3^, ^5^, ^11^ Factors such as advanced age, frailty, multiple comorbidities, admission outside the internal medicine department, longer in-hospital CPR duration, hospital type (general hospitals had higher mortality than tertiary general hospitals), and higher total hospital bed numbers reduce the chances of survival and good neurological outcomes.^2^, ^12^, ^13^, ^14^ However, recent progress in code response systems, early detection, and post-resuscitation care has led to modest improvements in survival rates since the late 2010s.^15^

In Thailand, the largest national database from the 2010 fiscal year included data on 17,813 older patients, with a discharge survival rate of 28.77%.^16^ However, these data are now outdated and may not reflect current patient demographics, comorbidities, or resuscitation practices. To date, there have been no recent national-level studies examining temporal trends in the prevalence of in-hospital CPR, survival outcomes, or factors associated with survival to hospital discharge among older adults in Thailand. This highlights a significant evidence gap, particularly with the rapid aging population and shifts in in-hospital resuscitation care. As a result, our goal was to analyze the trends of CPR in this population, their outcomes, and survival factors to hospital discharge using the latest national database from 2016 to 2024.

## Methods

### Population and study design

National data, sourced from the National Health Security Office (NHSO) in Thailand. The NHSO manages Thailand’s Universal Coverage (UC) program, which provides healthcare services to around 72% of the population. The Thai healthcare framework comprises public and private hospitals operating at primary, secondary, and tertiary levels. The Thai NHSO conducts an internal audit, overseen by a dedicated sub-committee, in addition to external audits. This process includes thorough financial management and quality control monitoring to ensure accurate diagnosis and medical intervention/procedure.^17^ The data included all inpatients aged 60 years or older who underwent in-hospital CPR procedures from January 1, 2016, to December 31, 2024. These procedures were defined by the International Statistical Classification of Diseases and Related Health Problems, 10th Revision, Thai Modification (ICD-10-TM), based on ICD-9-99.6, and the data were analyzed retrospectively. The dataset covered cardiac arrhythmias, categorized as atrioventricular and left bundle-branch block (ICD-10-I44), paroxysmal tachycardia (ICD-10-I47), atrial fibrillation and flutter (ICD-10-I48), other cardiac arrhythmias (ICD-10-I49), including ventricular fibrillation and flutter (ICD-10-I47), and unspecified cardiac arrest (ICD-10-I46), which includes cases of asystole and pulseless electrical activity (PEA). Cardiac arrhythmias were identified using ICD diagnosis codes recorded in the discharge summary. These codes reflect documented arrhythmias during hospitalization and do not necessarily correspond to the initial rhythm at the time of cardiac arrest. Additional data collected included patient age, sex, month and year of admission and discharge, length of hospital stay (LOS), primary diagnosis, relevant interventions or procedures (such as Coronary Artery Bypass Grafting (CABG) (ICD-9-36.10, 36.11, 36.12, 36.13, 36.14, 36.15, 36.16, 36.17, and 36.19), Intra-Aortic Balloon Pump (IABP) (ICD-9-37.61), Extracorporeal Membrane Oxygenation (ECMO) (ICD-9-39.65, 39.66), mechanical ventilation for 96 h or more (ICD-9-96.72), associated healthcare cost, and discharge status. Length of stay was determined by the entire duration of hospitalization, from admission until discharge or death. The database does not distinguish between pre-arrest and post-arrest hospitalization periods. Procedures were identified through discharge procedure codes documented during hospitalization. The database lacks details about when these procedures occurred in relation to the cardiac arrest event.

Data collection for all eligible patients was conducted through their summary discharge documentation. The study’s outcomes included age-adjusted prevalence and survival rates of older patients receiving in-hospital CPR to hospital discharge, normalized to a population of 100,000 Thai individuals. The analysis focused on survival rate at the time of subsequent CPR and associated factors of mortality at discharge.

### Definition of in-hospital cardiac arrest (IHCA)

IHCA was identified through procedure codes for CPR documented in the national discharge summary database. These codes indicate that CPR was performed during hospitalization; however, the database lacks detailed clinical information, such as pulselessness or the initial rhythm.

### Statistical analysis

Descriptive statistical methods were used to analyze the patients' demographic characteristics. If the data were normally distributed, we reported the mean and standard deviation; otherwise, we used the median and interquartile range (IQR). The prevalence rate per 100,000 individuals was evaluated across the entire population. Age-adjusted prevalence rates of in-hospital CPR were determined through the direct standardization method. Initially, age-specific prevalence rates were calculated for each predefined older adult age group. These rates were then weighted to reflect the proportional distribution of those age groups within the Thai national older population, which served as the reference standard. The final prevalence rates were reported per 100,000 people.^18^ The survival rate to hospital discharge per 100,000 individuals was calculated using a methodology analogous to that used for the prevalence rate. The hospital discharge survival rate was calculated as the number of individuals in each age group who underwent CPR, divided by the total number of individuals in that age group who received CPR. Associated factors of mortality at hospital discharge were examined using both univariate and multivariate analytical techniques. Factors with *p*-values < 0.20 in the univariate analysis were subsequently incorporated into a multivariate logistic regression model. A *p*-value < 0.05 was deemed indicative of a statistically significant difference, with 95% confidence intervals (CIs) reported to assess the strength of the association between the variables and mortality at discharge. This study obtained ethical approval from the Human Research Ethics Committee of the Institutional Review Board at Khon Kaen University (HE671169).

## Results

### Study population and characteristics

A total of 190,271 older patients who received in-hospital CPR from 2016 to 2024 are summarized in Table 1. Intervention/procedure use among these patients is shown in Table 2. The median age was 72years (IQR 66–79). The majority of patients were male and in the young-old age group (60–69 years). Cardiovascular disease was the most common primary diagnosis, followed by respiratory disease and gastrointestinal disease. A large number of patients were assigned discharge codes for unspecified cardiac arrest (including asystole/PEA); however, the dataset cannot determine if these codes reflect the initial arrest rhythm. The majority of the patients were hospitalized for more than 24 h (86.3%). The median hospital stay was 4 days (IQR 2–9). Patients who remained in the hospital for more than one week had higher survival rates than those who remained for 7 days or fewer. Higher survival rates to hospital discharge were observed among patients with healthcare costs exceeding 1530 USD.

**Table 1:** Baseline demographic characteristics of older patients who underwent in-hospital cardiopulmonary resuscitation (CPR) and survived to hospital discharge from 2016 to 2024.

**Variables**	**In-hospital CPR** ***n* = 190,271 patients** **(% of overall cohort)**	**Survival rates at hospital discharge** ***n* = 63,315 patients (33.3%)**
**Sex, *n* (%)**		
– Male	102,365 (53.7)	33,180 (32.4)
– Female	87,906 (46.3)	30,135 (34.3)
**Age group, year (%)**		
– 60–69	76,736 (40.3)	26,808 (34.9)
– 70–79	71,279 (37.5)	23,806 (33.4)
– ≥80	42,256 (22.2)	12,701 (30.1)
**Primary diagnosis, *n* (%)**		
– Cardiovascular disease	78,697 (41.4)	21,901 (27.8)
– Respiratory disease	40,282 (21.2)	16,946 (42.1)
– Gastrointestinal disease	16,518 (8.7)	4016 (24.3)
– Genitourinary disease	11,364 (6.0)	2476 (21.8)
– Neurological disease	10,400 (5.5)	6174 (59.4)
– Others	33,028 (17.2)	11,802 (35.7)
**Established cardiac arrhythmia, *n* (%)**		
– Heart block	4717 (2.5)	3406 (72.2)
– Paroxysmal tachycardia	18,009 (9.5)	9673 (53.7)
– Atrial fibrillation and flutter	35,650 (18.7)	14,998 (42.1)
– Ventricular fibrillation and flutter	6428 (3.4)	2686 (41.8)
– Unspecified cardiac arrest (included Asystole/PEA)	74,944 (39.4)	21,806 (29.1)
**LOS (day), *n* (%)**		
– ≤7 days	130,839 (68.8)	38,303 (29.3)
– 8–14 days	29,639 (15.5)	12,164 (41.0)
– >14 days	29,793 (15.7)	12,848 (43.1)
**Healthcare cost**		
– ≤50,000 baht (about 1530 USD)	125,143 (65.8)	33,918 (27.1)
– >50,000 baht (about 1530 USD)	65,128 (34.2)	29,397 (45.1)

Note: *n*; numbers of patients, LOS; length of stay (days), USD; United States Dollar.

**Table 2:** Intervention/procedure uses of older patients who underwent in-hospital cardiopulmonary resuscitation (CPR) and survived to hospital discharge from 2016 to 2024.

**Variables**	**In-hospital CPR** ***n* = 190,271 patients** **(% of overall cohort)**	**Survival rates at hospital discharge** ***n* = 63,315 patients (33.3%)**
– CABG	2176 (1.1)	1532 (70.4)
– IABP	4386 (2.3)	1716 (39.1)
– Pacemaker insertion	5101 (2.7)	3991 (78.2)
– ECMO	322 (0.2)	69 (21.4)
– Mechanical ventilation ≥96 h	37,944 (19.9)	13,372 (35.2)

Note: *n*; numbers of patients, CABG; Coronary Artery Bypass Grafting, IABP; Intra-Aortic Balloon Pump, ECMO; Extracorporeal Membrane Oxygenation.

### Prevalence rates of older patients receiving in-hospital cardiopulmonary resuscitation (CPR) from 2016 to 2024

**Fig. 1:**
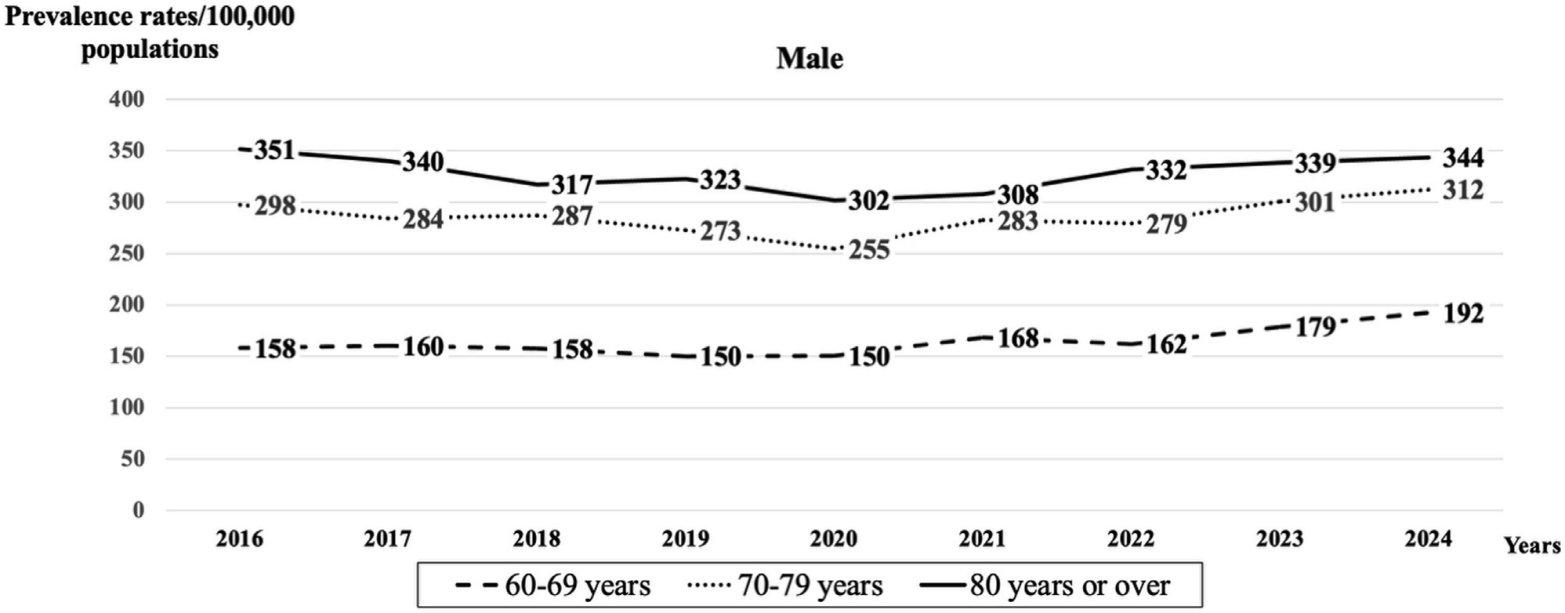
**Trends in age-adjusted prevalence rates, normalized to a population of 100,000 Thai males population with age groups 60–69, 70–79, and ≥80 years, utilizing in-hospital cardiopulmonary resuscitation (CPR) during the period from 2016 to 2024**.

**Fig. 2:**
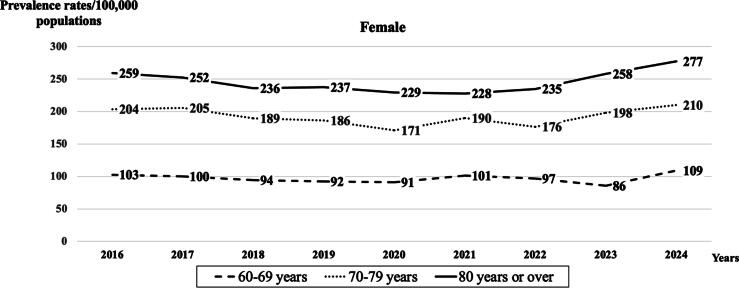
**Trends in age-adjusted prevalence rates, normalized to a population of 100,000 Thai females population with age groups 60–69, 70–79, and ≥80 years, utilizing in-hospital cardiopulmonary resuscitation (CPR) during the period from 2016 to 2024**.

Age-adjusted prevalence rates of older patients receiving in-hospital CPR, normalized to a population of 100,000 Thai males and females, were 221 and 147, respectively. Fig. 1, Fig. 2 display the age-adjusted prevalence rates for males and females, grouped by age: 60–69 years, 70–79 years, and 80 years or older. Females consistently showed lower prevalence rates than their male counterparts across all age groups and years. Notably, the oldest age group (80 years or over) had the highest prevalence rates across all years for both sexes. Additionally, both sexes experienced a decline in 2020, followed by a rebound and an upward trend from 2021 onward.

### Survival rates of older patients receiving in-hospital cardiopulmonary resuscitation (CPR) from 2016 to 2024

**Fig. 3:**
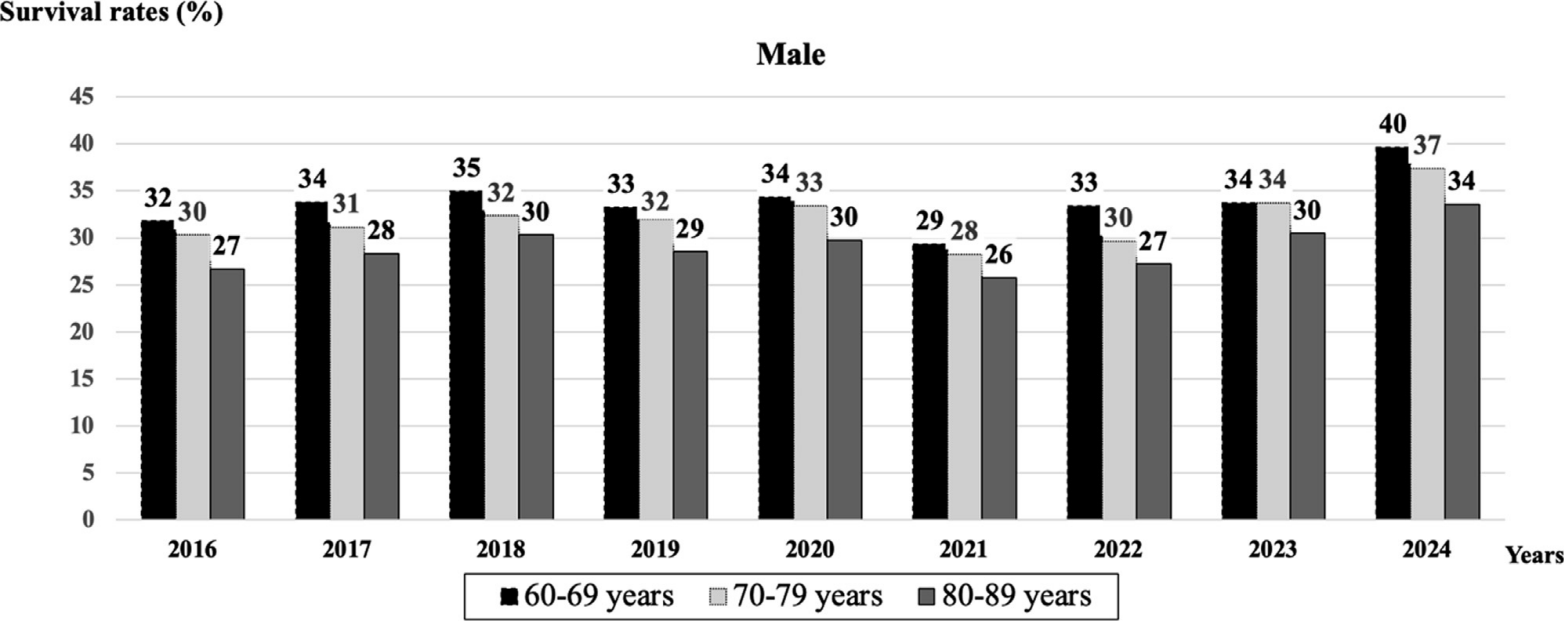
**Survival to hospital discharge (%) after In-Hospital CPR in males from 2016 to 2024**.

**Fig. 4:**
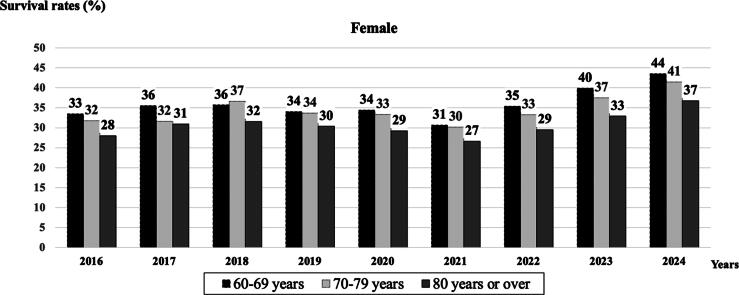
**Survival to hospital discharge (%) after In-Hospital CPR in females from 2016 to 2024**.

The overall survival rate to hospital discharge during the studied period was approximately one-third, with 32.4% in males and 34.3% in females (Table 1). The age-adjusted survival rate to hospital discharge per 100,000 population was 72 for males and 50 for females. Fig. 3, Fig. 4 present the survival rates (%) to hospital discharge stratified by age group and years for both sexes, respectively, from 2016 to 2024. In general, females exhibited higher survival rates to hospital discharge than males across all age groups over the years. Although the old-old age group experienced lower survival rates, this was an improvement compared to the past. The survival rates to hospital discharge showed a general upward trajectory from 2016 to 2024 for both sexes, notwithstanding a notable decline in 2021 followed by a robust recovery thereafter.

### Associated factors of death at hospital discharge

The factors associated with mortality at hospital discharge, as shown in Table 3, were identified using univariate analysis. All factors, including sex, age group, specific primary diagnoses, established cardiac arrhythmia, and various interventions and procedures, were included in the multivariate model (*p* < 0.2). The final model indicated that male gender, older age, primary diagnosis of cardiovascular disease, gastrointestinal disease, genitourinary disease, asystole/PEA, and receipt of IABP and ECMO were significantly associated with mortality. Conversely, primary diagnoses of respiratory and neurological diseases, presence of heart block, paroxysmal tachycardia, atrial fibrillation/flutter, ventricular fibrillation/flutter, and procedures such as CABG, pacemaker insertion, and mechanical ventilation for ≥96 h were inversely associated with mortality. The observed inverse relationship between extended mechanical ventilation (≥96 h) and mortality likely reflects survivor bias, as only patients who survived the early post-resuscitation period were able to receive prolonged ventilatory support. Although receiving procedures during hospitalization was associated with survival to discharge, the timing of these procedures relative to the cardiac arrest event cannot be determined.

**Table 3:** Factors associated with mortality at hospital discharge.

**Variables**	**Univariate analysis**		**Multivariate analysis**	
	**Odd ratios, 95%CI**	***p*-value**	**AOR, 95%CI**	***p*-value**
**Sex**				
– Female	1	−	1	−
– Male	1.09 (1.07–1.11)	<0.001	1.06 (1.03–1.08)	<0.001
**Age group (year)**				
– 60–69	1	−	1	−
– 70–79	1.07 (1.05–1.09)	<0.001	1.11 (1.08–1.13)	<0.001
– ≥80	1.25 (1.22–1.28)	<0.001	1.30 (1.27–1.34)	<0.001
**Primary diagnosis**				
– Cardiovascular disease	1.53 (1.50–1.56)	<0.001	1.33 (1.29–1.36)	<0.001
– Respiratory disease	0.62 (0.60–0.63)	<0.001	0.74 (0.49–0.55)	<0.001
– Gastrointestinal disease	1.61 (1.56–1.67)	<0.001	1.57 (1.50–1.64)	<0.001
– Genitourinary disease	1.85 (1.77–1.94)	<0.001	1.74 (1.65–1.83)	<0.001
– Neurological disease	0.32 (0.31–0.33)	<0.001	0.52 (0.49–0.55)	<0.001
**Established cardiac arrhythmia**				
– Heart block	0.18 (0.17–0.20)	<0.001	0.50 (0.46–0.54)	<0.001
– Paroxysmal tachycardia	0.39 (0.38–0.40)	<0.001	0.44 (0.42–0.45)	<0.001
– Atrial fibrillation and flutter	0.63 (0.61–0.64)	<0.001	0.66 (0.64–0.68)	<0.001
– Ventricular fibrillation and flutter	0.69 (0.65–0.72)	<0.001	0.98 (0.93–1.03)	0.45
– Unspecified cardiac arrest (included Asystole/ PEA)	1.37 (1.34–1.40)	<0.001	1.21 (1.19–1.24)	<0.001
**Intervention/procedure**				
– CABG	0.21 (0.19–0.23)	<0.001	0.30 (0.27–0.33)	<0.001
– IABP	0.77 (0.73–0.82)	<0.001	2.05 (1.91–2.21)	<0.001
– Pacemaker insertion	0.13 (0.12–0.14)	<0.001	0.24 (0.23–0.26)	<0.001
– ECMO	1.83 (1.40–2.39)	<0.001	5.18 (3.84–6.97)	<0.001
– Mechanical ventilation ≥96 h	0.90 (0.88–0.92)	<0.001	0.88 (0.86–0.91)	<0.001

Note: AOR; adjusted odds ratio, CI; confidence interval, CABG; Coronary Artery Bypass Grafting, IABP; Intra-Aortic Balloon Pump, ECMO; Extracorporeal Membrane Oxygenation.

## Discussion

### Trends in the prevalence rates of older patients receiving IHCA and their characteristics compared to previous studies

This national data showed that prevalence rates of older patients undergoing in-hospital CPR were higher among males and in older age groups during 2016–2024. Both sexes experienced a decline in 2020. Compared with the nationwide cohort study in South Korea for 2010–2019, the prevalence rates were 83.5/100,000 adults in 2016 and gradually increased to 92.1/100,000 adults in 2019.^2^ The Korean study's mean age was 70 years, unlike ours, which reported prevalence rates of 221 and 147 per 100,000 for older men and women, respectively, and higher in older groups. Differences likely stem from methodology: this study used rates per 100,000 for those 60 years or older, while the Korean study included all ages, likely resulting in higher figures. Other possible explanations included differences in cultural norms regarding the preference for life-prolonging treatments and the integration of palliative care. In South Korea, there are comprehensive end-of-life laws, widespread advance care planning, and well-established palliative services that likely lead to fewer CPR attempts among frail older adults.^19^, ^20^ Other reports are limited in direct comparison with our study because some use different denominators, such as 'per 1000 admissions’, which reflect hospital activity, whereas our study used 'per 100,000 population' to measure the burden in the underlying population.^16^

Cardiovascular disease was the leading diagnosis among older CPR patients, aligning with prior studies. For example, a U.S. study reported 50–60% of cardiac arrests due to cardiac disorders. Most patients had unspecified arrests, including asystole/PEA, matching findings that most had non-shockable rhythms like asystole PEA.^1^, ^16^, ^21^ The typical length of stay was usually under a week, similar to the previous report in Thailand.^16^ Limited regional data, especially on older post-in-hospital cardiac arrest patients. For example, a small Lebanon study reported a mean hospital stay of 7.76 days (SD 10.2) among older patients survivors.^22^

### Trends in the survival rate to hospital discharge of older patients receiving IHCA compared to previous studies

The survival rate to hospital discharge for older patients after in-hospital CPR was about one-third, slightly above Thailand's 2010 study reporting 28.77% rate.^16^ The survival rate to hospital discharge has increased over the past three years (2022–2024) across all age groups. These findings may result from earlier detection and monitoring technologies, improved post-resuscitation and intensive care,^15^, ^19^, ^20^, ^23^, ^24^ changes in patient selection for CPR, and advancements in hospital quality and data accuracy.^15^, ^25^, ^26^ Interestingly, the figure was higher than the 18.7% reported in a systematic review^13^ and in several studies, ranging from 17.0% to 28.5%.^2^, ^3^, ^5^, ^11^ The likely explanation is the difference in case definition: Thai datasets include procedure codes for CPR at any admission point, while Western registries only count cardiac arrests needing chest compressions and resulting in loss of pulse, leading to lower survival estimates.^11^ Coding bias and discharge definitions can overestimate survival, as Thai hospitals record “discharge alive” for transferred patients, regardless of short-term survival. In the Thai administrative database, patients are labeled as “discharged alive” if they leave the initial hospital alive, which includes transfers between hospitals or discharge to home. However, the database does not record deaths that happen after transfer or soon after discharge. On the other hand, many registry-based studies measure survival at the time of final hospital discharge. These structural differences can affect the reported survival rates.^11^, ^27^

### Associated factors of death of older patients receiving IHCA compared to previous studies

This study found that higher death rates at discharge were associated with male gender, older age, and primary diagnoses such as cardiovascular, gastrointestinal, and genitourinary diseases, while respiratory and neurological diseases were linked to lower mortality. Men had higher hospital discharge mortality after in-hospital cardiac arrest than women. Possible reasons include biological differences like cardioprotective effects in females, but these are speculative and untestable in this study. Alternative explanations involve patient case mix differences, such as more cardiovascular disease or severity among males. Variations in post-resuscitation care, treatment, or end-of-life decisions might also contribute.^28^ Sex-based differences in DNAR orders, withdrawal of life-sustaining therapy, or discharge practices may influence outcomes but couldn't be evaluated through administrative data. Compared to earlier reports on out-of-hospital cardiac arrest, findings on sex-based survival differences vary across studies, suggesting factors like arrest setting, patient selection, and health-system differences could influence results.^29^, ^30^ The association should be viewed as descriptive, not causal. More research with detailed clinical data is needed to understand sex-based disparities in IHCA outcomes among older adults.

Increasing age was another independent factor of death upon hospital discharge, consistent with several studies. These likely results from age-related physiological decline, a higher burden of comorbidities, the predominance of non-shockable rhythms, and limited availability of post-resuscitation interventions.^2^, ^3^, ^13^ Patients with cardiovascular, gastrointestinal, or genitourinary diseases had higher mortality after in-hospital cardiac arrest, likely due to organ dysfunction, reduced physiological reserve, and metabolic issues that impair resuscitation and recovery arrest.^1^, ^31^, ^32^ Patients with chronic diseases often face multi-organ damage, lowering resilience and increasing vulnerability to ischemia–reperfusion injury, leading to worse outcomes post-treatment.^12^ However, this study found lower discharge mortality linked to respiratory and neurological diagnoses, indicating that closer monitoring, prompt CPR, and addressing reversible causes like hypoxia or airway obstruction can improve survival after in-hospital cardiac arrest.

This study indicates that before cardiac arrest, unspecified cardiac arrest (including asystole/PEA) was linked to higher mortality, while heart block, tachycardia, atrial fibrillation/flutter, and ventricular fibrillation/flutter correlated with lower death rates. These findings align with reports classifying asystole/PEA as a non-shockable rhythm with poorer survival than shockable rhythms like ventricular tachycardia or fibrillation. Asystole and PEA usually stem from severe conditions such as hypoxia, acidosis, or multi-organ failure, rather than reversible arrhythmias. They are less responsive to defibrillation, lowering resuscitation success. Heart block patients often have reversible causes like medication effects or electrolyte issues, and may be treatable with devices. Consequently, cardiac output can be quickly restored.^7^, ^16^, ^33^

Regarding post-resuscitation treatments, the association between IABP or ECMO use and an increase in in-hospital mortality warrants careful consideration. These results probably reflect confounding by indication, as IABP and ECMO are more often used in patients with severe instability or refractory shock after IHCA. Evidence for IABP's benefit in cardiac arrest is limited, and prior studies show higher mortality in recipients, likely due to greater baseline severity, not the device's effect.^34^ Similarly, while ECMO has been demonstrated to improve survival in carefully selected patients by providing temporary circulatory support and enabling treatment of reversible causes, its effectiveness heavily depends on patient selection, downtime, and comorbidity burden.^35^, ^36^ Due to limited clinical severity data in our dataset, we can't fully account for these factors. Thus, associations should not imply IABP or ECMO increases mortality, but indicate severe post-arrest illness. Future research with detailed clinical data is needed to clarify their impact.

Procedures such as CABG and pacemaker insertion were associated with lower mortality in this study. The results aligned with earlier reports, suggesting that the mechanism may involve improved myocardial perfusion, better ventricular function, and more attentive cardiac monitoring.^6^, ^37^ In this study, mechanical ventilation exceeding 96 h was associated with lower mortality, a finding that contrasts with existing data.^6^, ^16^ Differences may reflect successful stabilization and support instead of a poor prognosis. Older patients surviving the initial cardiac arrest phase often need extended ventilatory support for recovery, lung protection, or weaning, indicating stability beyond the high-mortality period. Prolonged ventilation may also suggest selection for continued treatment based on recovery potential, lowering the overall mortality rate, which reflects survivor bias.^15^

### Implications for clinicians and policymakers

These findings have important clinical and public health consequences. Higher IHCA rates among older adults and males highlight the need for targeted prevention, such as close monitoring of high-risk older patients. Mortality trends demonstrate the benefits of proactive management for cardiovascular, gastrointestinal, and genitourinary conditions, while lower mortality in respiratory and neurological cases may indicate more reversible issues. Improved outcomes are associated with shockable rhythms and treatments like CABG, pacemakers, and prolonged ventilation, underscoring the importance of early cardiac monitoring and personalized care. Variations in coding, population data, and cultural attitudes toward life support may explain international differences, highlighting the need for standardized reporting and ongoing evaluation of outcomes in aging populations.

### Limitations of the study and future research

This study had some limitations. First, its retrospective design limited the analysis to certain factors and outcomes, such as ICU admission, medication use, disease severities, details of Do-Not-Attempt-Resuscitation (DNAR) status, and the timing of interventions or procedures, whether intra- or post-cardiac arrest, functional and cognitive recovery, quality of life after cardiac arrest, post-hospitalization costs, and other long-term effects. Thus, residual confounding may still be present, and the multivariable analyses should be viewed as revealing associations at the population level rather than establishing causal effects or predicting individual outcomes. Second, relying on ICD-10 coding for patient identification could result in some patients being missed due to physician misclassification, including the potential inclusion of brief resuscitative efforts without sustained pulseless cardiac arrest. Nonetheless, this approach has been widely used in national epidemiological studies, and any misclassification is likely to be non-differential. Third, the IHCA rate per 1000 admissions could not be presented because the database only contained records of IHCA patients, not total hospital admissions. Future research should focus on collecting the missing data noted as a limitation and ensuring complete admission data to obtain more consistent rates. Fourth, the administrative dataset lacks time-specific details about procedures related to the cardiac arrest event. Consequently, it is not possible to determine whether procedures occurred before or after the arrest. Relationships between procedures and survival outcomes might reflect survivorship bias or reverse causation and should not be seen as proof of causality. Fifth, survival to discharge was defined as surviving through discharge from the initial hospital stay. The database does not support follow-up for patients transferred elsewhere or mortality tracking after discharge. As a result, the reported survival rates may not reflect the final hospital discharge status or short-term outcomes. Additionally, cultural preferences for end-of-life care at home could lead to an underestimation of in-hospital death rates. Lastly, relying on administrative discharge data limits our ability to accurately identify the initial arrest rhythm. Diagnosis codes for arrhythmias may reflect events that occurred at any point during hospitalization and should not be interpreted as the rhythm at the onset of cardiac arrest. Consequently, the proportion of shockable versus non-shockable initial rhythms cannot be precisely determined in this study.

## Conclusions

This study from 2016 to 2024 examined the prevalence rates and associated factors of mortality among older patients with IHCA. CPR rates were higher among males and older groups. Cardiovascular disease was the most common primary diagnosis. Discharge codes primarily indicate non-shockable arrhythmias; however, the initial arrest rhythm cannot be identified in administrative data, underscoring the need for improved early detection of clinical deterioration. Overall survival to hospital discharge was about 33%, which was better than past Thai data. Mortality was associated with male sex, older age, certain primary diagnoses, use of ECMO, and IABP. Outcomes were worse with asystole or PEA but improved with CABG, pacemakers, or prolonged ventilation. These findings underscore the importance of risk stratification, early detection, tailored prevention for high-risk older inpatients, and integrating geriatric-focused care in hospital cardiac arrest treatment.

## CRediT authorship contribution statement

**Manchumad Manjavong:** Writing – review & editing, Writing – original draft, Methodology, Investigation, Conceptualization. **Panita Limpawattana:** Writing – review & editing, Writing – original draft, Project administration, Methodology, Investigation, Data curation, Conceptualization. **Jarin Chindaprasirt:** Writing – review & editing, Writing – original draft. **Watsamon Inyu:** Writing – review & editing, Writing – original draft, Conceptualization. **Poonchana Wareechai:** Writing – review & editing.

## Funding

There is no funding.

## Declaration of competing interest

The authors have no potential conflicts of interest to declare with respect to the authorship of this manuscript.

## Data Availability

The data that support the findings of this study are available from the corresponding author, PL, upon reasonable request.
